# Response to Taïeb’s “Evidence-Based Moisturizer Selection for Atopic Dermatitis”

**DOI:** 10.1016/j.jdin.2025.05.001

**Published:** 2025-05-17

**Authors:** Nadia Shobnam, Grace Ratley, Jordan Zeldin, Manoj Yadav, Ian A. Myles

**Affiliations:** Epithelial Therapeutics Unit, National Institute of Allergy and Infectious Diseases, National Institutes of Health, Bethesda, Maryland

**Keywords:** atopic dermatitis, ceramide, emollients, sphingolipids

To the Editor:

The authors begin their report alleging that support for ceramide-based topicals is unevidenced by citing a meta-analysis from 2017 which does not contain any study of ceramide-based topicals published after 2014 and only assessed one named ceramide-containing product, EpiCeram.^1^ Although citations may be limited by the journal, we suggest the authors update their literature search to include studies from the most recent decade; these show that although paraffin- and ceramide-based emollients have equivalent impacts on barrier function, ceramide-based approaches are superior at improving symptoms.^2^ Furthermore, deficiencies in ceramides and sphingolipids are routinely identified in patients with atopic dermatitis and are the most consistent early-life predictors of atopic dermatitis risk.^3^ Therefore, while the molecular diversity of an emollient’s ingredients may impact its efficacy, categorical replacement of ceramides and sphingolipids holds the potential for disease-specific supplementation.

The authors next dismiss the concerns regarding excipient-induced dysbiosis as they are based solely on “*in vitro* data of the so far poorly validated dysbiosis criterion.”^1^ Simply reading the abstract, text, or figures of the cited publication would have revealed that our report also included both mouse and human studies.^3^ Although our human data are only proof-of-concept, the ability of topical products to influence the survival and metabolism of skin microorganisms is well established^4^^,^^5^ and unlikely to be restricted to the petri dish. The authors suggest that product assessments should include “established pharmacopeial standards to guarantee the microbial safety of moisturizers.”^1^ This mistakenly centers “safety” on the moisturizer rather than the human. Indeed, removal of preservatives from topical products would risk contamination. However, this should prompt the realization that these preservatives are functioning as prophylactic antibiotics for the emollient, which present risk for dysbiosis, contact dermatitis, and irritation.

Their final claim is that their group’s guidelines represent a level of evidence that is superior to double-blind clinical trials. They use this fabricated hierarchy to suggest a preference for moisturizing with “5% urea (carbamide)” and/or “two glycerol-containing creams.”^1^ However, their endorsement of three specific products is inconsistent with the meta-analysis they cited, which concludes “we did not find reliable evidence that one moisturizer is better than another.” The only other citation is an unreviewed abstract summarizing a meeting, hardly considered “based on a hierarchy of data from authorized clinical research.”^1^ We cannot assess the full ingredient list of the topicals they suggest beyond glycerol and urea but note that urea compounds tend to skew the skin’s pH higher than normal and possess *in vitro* antimicrobial potential. However, the one product with 5% urea tested in our method, CeraVe Healing Ointment, did not impact bacterial survival.^3^

We do, however, agree that patient outcomes should be the primary metric for any emollient. We therefore eagerly await the authors’ results from a controlled clinical trial, transparent reports of their products’ excipients, and their assessments of the products’ impact on the host and microbiome. Until then, we adhere to our prior statement that, “it remains controversial to recommend one emollient over another.”^3^

## Conflicts of interest

None disclosed.
